# Integrating economic and evolutionary approaches to polygynous marriage

**DOI:** 10.1017/ehs.2022.49

**Published:** 2022-10-26

**Authors:** Siwan Anderson, Chris Bidner

**Affiliations:** 1 Vancouver School of Economics, University of British Columbia, Vancouver, Canada; 2 Department of Economics, Simon Fraser University, Vancouver, Canada

**Keywords:** Marriage, family, polygyny, competition, efficiency

## Abstract

We outline the potential for integrating economic and evolutionary approaches to marriage and the family. Our broad argument is that the approaches share a concern for *competition*. Evolutionary scholars are concerned with the fitness consequences of competition and economists are centrally concerned with the nature of competition: how the allocation of scarce resources is mediated by potentially complex forms of social interaction and conflicts of interest. We illustrate our argument by focusing on conceptual and empirical approaches to a topic of interest to economists and evolutionary scholars: polygynous marriage. In comparing conceptual approaches, we distinguish between those that emphasise the physical environment and those that emphasise the social environment. We discuss some advantages of analysing marriage through the lens of competitive markets, and outline some of the ways that economists analyse the emergence of rules governing the family. In discussing empirical approaches to polygynous marriage, we describe how a concern for informing contemporary policy leads economists to focus on the consequences of polygyny, and in particular we describe some of the ways in which economists attempt to distinguish causal effects from selection effects.

## Graphical abstract

**Figure:**
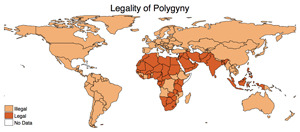

**Social media summary:** There is great scope for integrating economic and evolutionary approaches to the study of marriage and the family

## 1. Motivation

An interest in understanding marriage and the family is shared by both economists and evolution-based scholars from a variety of disciplines including biology, anthropology, psychology and sociology. In this paper we propose ways in which the perspectives and methods of economics can be usefully integrated with evolutionary approaches. In general terms, an evolution-based explanation rests on three Darwinian components: variation, competition and inheritance (Mesoudi, 2011). Economics has great scope to contribute to evolution-oriented scholarship because it is the study of how scarce resources are allocated. It is, in essence, the study of the competition component. In particular, economics is squarely concerned with understanding social interactions and conflicts of interest. The scope for economics to contribute is particularly large in the context of *human* behaviour because ‘the complexity and variety of social interactions among human individuals is without parallel …, and conflicts of interest are also diverse and complex to an unparalleled degree’ (Alexander, 1987). Complexity calls for transparent and precise analysis of competition. Diversity calls for a broad appreciation for the range of ways in which competition can play out. Economics answers both of these calls.

Economists are concerned with informing contemporary policy issues. The desire to avoid unintended consequences of policy naturally moulds a methodology designed to cut through the complexity presented by social interactions. On the conceptual front, this means utilising formal mathematical models. Doing so forces analysts to describe the competition component in terms that are precise and familiar to other analysts. At a minimum, this means spelling out who the agents are, what actions they can take, what their preferences are, and some notion of equilibrium (i.e. some sense in which agents’ choice of actions are mutually consistent). On the empirical front, this means taking pains to identify causal effects and exploiting certain types of data and regression techniques to rule out confounding factors.

The remainder of the paper elaborates upon the claim that there is great potential for integrating economic and evolutionary perspectives on the family. This claim applies broadly, but we shall illustrate it by focusing on but one issue of common concern to economists and evolutionary scholars: polygynous marriage (polygyny hereafter; see Fortunato, 2015 for a discussion of polygynous marriage vs. polygynous mating). We begin with conceptual approaches in Section 2, where we do our best to place all explanations, economic and evolutionary, on common ground by offering an explicit description of each theory's competition component. We organise this illustration by separating explanations that emphasise the physical environment (distribution of resources) from those that emphasise the social environment (imposition of rules). We illustrate three broad ways in which economics can contribute to evolutionary perspectives on the family:

1. Many relevant interactions (e.g. finding marriage partners) are indeed complex, involving a large number of heterogeneous individuals simultaneously attempting to meet their own objectives. Competitive markets are a powerful lens, widely used by economists, through which to organise such complexity.
2. Human interaction is often subject to rules (e.g. against polygyny), and these rules can emerge as attempts to improve efficiency: i.e. limit the extent to which resources are wasted. Economists have identified a broad range of settings in which inefficiency is likely to arise.
3. Rules can also emerge as the result of conflict between social groups with varying degrees of power (e.g. the wealthy and the poor). Economics encompasses fields, most notably political economy, that are centrally concerned with the dynamics and use of power.

We follow this up with a discussion of empirical issues in Section 3. Here we emphasise the focus on distinguishing between causality and selection effects. We discuss conclusions in Section 4.

## 2. Polygyny: conceptual approaches

Polygynous marriage – whereby males can have multiple wives – is a complex practice exhibiting important variation across societies (Lawson & Gibson, 2018; White, 1988). Rather than attempt an exhaustive survey that does justice to this variety (Fortunato, 2015; Schacht & Kramer, 2019; White & Burton, 1988), our goal here is to strip the practice down to its core features in order to best identify the key differences in perspective taken by economic and evolutionary approaches. Abstracting in this way is not without cost, an issue we return to in Section 3, but it does offer a useful starting point.

We divide approaches to polygnous marriage into two broad categories: those that emphasise the physical environment and those that emphasise the social environment. Applied to polygyny, this categorisation is similar to the notions of ‘ecologically imposed monogamy’ and ‘socially imposed monogamy’ from Alexander et al. (1979). We opt for more general terms since the distinction is useful far beyond polygyny, and because we want to be be able to clearly distinguish the insights of Alexander et al. (1979) from that of others. By ‘physical environment’ we primarily mean the availability and distribution of resources. The core issues here will be familiar from biology, and thus there is clear scope to draw inspiration from the mating patterns of non-human species. Yet humans have additional capacities that allow for more sophisticated forms of exchange. By ‘social environment’ we primarily mean the set of rules that humans superimpose upon their interactions in the physical environment. Here the core issues are quite different and less directly related to biology.

Naturally, these two approaches are interrelated. The distribution of resources is surely sensitive to prevailing rules, and the set of rules that emerge is surely sensitive to the distribution of resources. Yet we consider it useful to separate the approaches since they raise distinct issues. The nature, availability and distribution of resources are central when considering the physical environment, whereas sources of resource wastage and power dynamics are central when considering the social environment. In addition, the two approaches ask distinct questions. For instance, consider attempts to understand monogamy. The physical environment approach seeks conditions under which competition leads to males and females having no more than one spouse. In contrast, the social environment approach seeks conditions under which a society has come to be bound by rules against polygyny.

### 2.1. Physical environment

#### 2.1.1. Early evolutionary approaches to polygynous marriage

Early evolution-based approaches to polygynous marriage (e.g. Borgerhoff Mulder, 1990, 1992; Chisholm & Burbank, 1991) take inspiration from classic biological models of polygynous mating of non-human species. These classic models, although not formalised in detail, have conceptually simple competition components. For our purposes, we group the main classic models under two broad categories; ‘female choice’ and ‘male choice’.

Female choice models include the polygyny threshold model (Orians, 1969), resource defence polygyny and male dominance polygyny (Emlen & Oring, 1977). The competition component could be described as follows. Males are heterogeneous with respect to their ability to maintain control over resources whereas all females are identical. Females choose which male to mate with, and the male's resources are divided equally among all females that choose him. Equilibrium occurs when no female would choose a different partner given the choices made by all other females. The key trade-off facing females is that whilst wealthier males are more attractive all else equal, they also attract more females and thus those resources must be shared more widely. A central conclusion is that polygyny arises from inequality among males.

Male choice models are less prominent, but include female (or harem) defence polygyny (Emlen & Oring, 1977). The competition component is similar to female choice, except that males compete directly for females, as opposed to the resources that would attract them. For instance, Betzig (1986/2017) emphasises ‘the Darwinian hypothesis that individuals will exploit positions of strength in resolving conflicts in their own interest, and that ultimately they will seek reproductive rewards’. The competition component could be described as follows. Males secure access to a given female with a probability proportional to how hard they compete for her relative to other males. Males choose how hard to compete for each female, and an equilibrium arises when no male wishes to change their efforts given the choices of all other males. The trade-off for males is that more intense effort depletes resources but raises the probability of securing females. Here too polygyny is promoted by male heterogeneity, and in particular by the existence of powerful males in despotic societies (see Betzig, 1986/2017).

Whilst these classic models offer transparent accounts of the competition underlying polygyny and thus a useful lens, they risk being too simple and especially so when applied to humans. First, both sides, male and female or those acting on their behalf, actively participate in partner choice. Of course, the study of non-human mating systems has greatly advanced since this classic work, for instance by incorporating mutual choice (e.g. Courtiol et al., 2016), but this work is typically not concerned with polygynous mating and therefore has been less influential as a basis for understanding polygyous marriage. Second, and more importantly for our purposes, both sides can actively influence their attractiveness via competing offers of resources.

#### 2.1.2. Economic approaches to polygynous marriage

In understanding marriage, economists see stronger analogies to competitive markets than to the mating behaviours of other species. In the broadest possible terms, a market, including those studied by biologists (e.g. Nöe & Hammerstein, 1995; Nöe et al., 2001), is the setting in which members from two classes of agents – buyers and sellers, workers and firms, males and females, etc. – interact for mutual benefit. In order to gain analytical tractability over such settings, economists utilise the fiction of a *competitive* market (for an extensive overview of other approaches see Chade et al., 2017). This is a specific idealisation in which there is a commonly observed ‘price’, which all agents take as given. Importantly, the notion of a price presupposes that both sides are able to offer transfers to the other in an attempt to attract partners (in contrast to models based on Gale & Shapley, 1962, such as Bergstrom & Real, 2000). This price adjusts until all desired trades can be executed (supply equals demand). This modelling device offers an elegant way to abstract from the complexities that generally arise from a game theoretic analyses involving large populations.

The classic competitive model of marriage in economics was introduced by Becker (1973, 1974, 1991; see also Grossbard-Shechtman, 1993; Grossbard, 2015). The model considers a competitive marriage market with males and females that are deciding upon potential partners. In the simplest version, a household is formed by one male and at least one female. Potential household members anticipate that, once married, they will produce a ‘household good’. The quantity of the household good varies with the characteristics of household members, and this total can be divided between them. Preferences are simple: all individuals, male or female, prefer more of the household good to less. Unlike the classic biological mating models described above, the allocation of the household good across household members is determined by market competition, not by a fixed rule (e.g. equal sharing). The market works by proposing a ‘price’ for each female. This price is interpreted as the amount of the household good that she is to be allocated, and the household's male is allocated the remainder after each female claims her share. To simplify the explanation, suppose that the females were identical so that there is a single price in the market. Given this price, each male decides how many wives they want to marry and each female decides whether they want to marry. This price adjusts so that the total number of females desired by males (demand) equals the total number of females willing to marry (supply). That is, an equilbrium here describes not only who marries whom but also how each household so formed divides the household good among members.

Nothing of substance changes if females varied in their characteristics. Each female has a particular *type* and each such type has an associated ‘price’. These prices adjust so that the total demand for each type of wife equals the total supply of each type of wife (see Rosen, 1974 for the general theory and Peters & Siow, 2002 for the application to marriage). Similarly, the fiction of a ‘household good’ is convenient but unnecessary. What is important is that each group has flexibility in allocating the surplus utility that it creates. A female's price is then interpreted as the utility that she commands. As with the classic evolutionary models, this basic version predicts that polygyny is promoted by inequality among males and female-skewed sex ratios. Yet the extra effort in the modelling process is useful because it illuminates so much more than this.

*Insights from a competitive market perspective*: The competitive market perspective offers at least three broad sorts of insights. The first is that the analysis also tells us how the gains from marriage are allocated between spouses. This is important when males and females have conflicting preferences over how the household should operate (e.g. how many resources to allocate to offspring). Such conflict is pervasive, and likely has an evolutionary basis (see Parker, 1979, 2006; Brown et al., 2009; Scelza, 2013; Jennions & Fromhage, 2017). The way in which the physical environment affects the resolution of this conflict is of central importance, e.g. for child development outcomes, yet cannot be analysed with models where household resources are allocated in a fixed manner. To be sure, economists have developed a range of other approaches to spousal bargaining over household resources (see Browning et al., 2014; Baland & Ziparo, 2018 for overviews), many of which are well-known to human behavioural ecologists (e.g. Pollet & Nettle, 2008; Gurven et al., 2009), but these models apply once the marriage has formed. In contrast, in the competitive approach the bargaining process is used in order to attract spouses.

Moreover, understanding how the gains from marriage are allocated helps us make sense of a range of related issues. There is an immediate relationship to the direction and magnitude of marriage payments such as brideprice and dowry. Such payments may be crucial in achieving the market-determined allocation of the household good. This is especially so in situations where spouses cannot make binding agreements at the time of marriage about how the household good will be divided in the future (Anderson & Bidner, 2015). In such cases marriage payments act as an upfront transfer that offsets foreseeable changes in how the household good will be divided. Marriage payments introduce other considerations, many of which are of interest to evolutionary anthropologists (e.g. Dickemann, 1979; Gaulin & Boster, 1990; Ross et al., 2016). For instance, as Bergstrom (1996) points out, ‘males can use the bride prices received for their female relatives to purchase brides for themselves’. This gives male relatives a stake in the bride's ‘market return’, which has implications for how much parents invest in daughters, the efficacy of public policies such as the expansion of schooling opportunities (Ashraf et al., 2020) and the strength of norms of female seclusion (since females command a higher ‘price’ if they can offer greater paternity certainty). Appreciating the relevance of negotiations over marital surplus helps us understand the contractual nature of marriage. For instance, promises of future household surplus may not be credible without the binds of a marriage contract. This is especially true if the wife foresees a weaker bargaining position in the future because of a household division of labour in which the husband is the primary earner (Anderson et al., 2021).

The second broad insight delivered by a market perspective concerns female heterogeneity. The plausibility of the female choice model is reduced when females vary in quality. For example, what if the highest quality male finds that he has attracted a group of the lowest quality females? For non-human species, such as the birds that inspired the polygyny threshold model, this may just be bad luck for the male. However, this seems implausible for humans given our ability to negotiate. In contrast, female heterogeneity is easily handled by Becker's market model. Morevover, female heterogeneity is indispensable if we want to understand who marries whom – e.g. do high-quality males tend to marry high-quality females? This issue is not straightforward when marital surplus is divisible since lower quality partners need not be less attractive if they offer sufficiently large transfers (e.g. see Legros & Newman, 2007). Furthermore, the issue plays an important role in understanding the evolutionary fitness of traits such as altruism (e.g. see Alger & Weibull, 2013).

Even if females are equally fecund, female heterogeneity is particularly relevant in settings where parents strive for quality over quantity in their offspring. Indeed the extent of female heterogeneity, as with male heterogeneity, is a crucial factor in determining the extent of polygyny. Gould et al. (2008) shows that, unlike the male counterpart, greater inequality among females *reduces* the extent of polygyny. The authors use this insight to argue why polygyny has declined in rich countries despite increases in male inequality. The argument is that females are heterogeneous in their ability to raise high-quality offspring and this heterogeneity is effectively amplified as modernity raises the return to child quality. Greater heterogeneity among females then acts to reduce polygyny as males begin to prefer quality over quantity in wives.

The third broad insight, although not limited to a market perspective, is that extensions are readily accessible. Although many features of the market model appear unrealistic or incomplete, the formality and generality provide a common foundation that allows us to add features or relax assumptions in a transparent way. For instance, if polygyny is promoted by inequality among males then it is useful to understand where this inequality comes from. It is particularly insightful if male inequality is itself, at least partially, a *consquence* of polygyny. Without a model, it is prohibitively difficult to grasp, for instance, how polygyny, wealth inequality and economic development co-evolve. Lagerlöf (2005) embeds polygyny in a model of long-run economic development, producing a tractable means to understand how polygyny and heterogeneity are jointly determined. In his model, inequality among males arises because of inequality of landholdings, but the wealthy drive technological progress owing to their surplus resources which allow for ‘thinking’. Technological progress eventually makes land relatively less important in income generation, and this tends to equalise wealth. Polygyny declines as a result.

Rather than adding more structure, it is often useful to relax the model assumptions. For instance, the basic Becker model is concerned with a marriage market that operates as if at a single point in time. This is a useful abstraction, but it does not allow insight into the effect of phenomena such as population growth. This is relevant for polygyny since males can have multiple wives, even if the sex ratio is unity at each age and there is no inequality among males, if they marry younger women and there is population growth (Neelakantan & Tertilt, 2008; Tertilt, 2005). We briefly discuss similar extensions in Section 4.

#### 2.1.3. Recent evolutionary approaches to polygynous marriage

Relative to the classic evolutionary models, modern evolutionary approaches to polygynous marriage utilise more mathematically sophisticated (typically game theoretic) formal models, and this has allowed researchers to analyse more nuanced perspectives on polygyny. Such models extend the classic biological models in important ways, and in particular feature mutual choice. Yet there remains scope for incorporating elements from competitive markets. We discuss two models that showcase the promise of expanding the competition component in evolutionary settings.

For instance Fortunato and Archetti (2010) consider how the important issue of intergenerational wealth transmission interacts with partner choice. The competition component considers homogenous males and homogeneous females. Each male is characterised by how many wives they seek as well as how to transmit wealth across generations (give to wife's offspring or to sister's offspring). Females are characterised by how many husbands they seek; effectively, the degree of paternity certainty they offer. There is mutual choice in the sense that the assignment of marriage partners is sensitive to the strategies of both males and females. There is no conflict over household resources because the only allocation decision is intergenerational and under the full control of males. Matching is random, with agents being assigned either their desired number of spouses or none. That is, attempts to gain partners via competing offers of resources (or promises of a particular intergenerational transmission pattern) is ruled out by assumption. The relative frequency of types in the population depends on relative inclusive fitness, and equilibrium arises when the type frequencies are such that no rare ‘mutant’ type is able to achieve a greater inclusive fitness. Monogamy can arise as a result of a population of males that give their resources to their wife's offspring if and only if the wife offers high paternity certainty, and females offer high paternity certainty if and only if husbands are monogamous. This requires the male contribution to offspring to be sufficiently large, and the intuition is that a mutant male that chooses to seek many wives will suffer in terms of inclusive fitness since his wives’ offspring are less likely to be his.

As a second example, Ross et al. (2018) offer a closer examination of the relationship between polygyny and male inequality. The competition component of their model, more fully described in Oh et al. (2017), considers homogeneous females and heterogeneous males endowed with rival wealth (e.g. land) and non-rival wealth (e.g. genes, local knowledge). Rival wealth per wife decreases in the number of wives whereas non-rival wealth does not. Importantly, males also experience diminishing fitness returns to additional wives for reasons unrelated to the sharing of rival wealth. The model features mutual choice, with males choosing the maximum number of wives to marry and females choosing their preferred male given the choices of other females. The approach is game theoretic, analysing Nash equilibrium marital assignments, rather than taking a competitive market approach. The issue of how the allocation of household resources responds to environmental conditions does not arise because of an assumption that males gain no payoff from consuming their rival wealth, leading them to willingly give it all to their wives in equal proportions. There is an exogenous brideprice which does not respond to market conditions and does not go to the bride in any case. The formal analysis adds insightful nuance to the claim that ‘male inequality promotes polygyny’, demonstrating that it matters how one measures inequality (and polygyny). For instance, males can be unequal because a large minority are somewhat wealthier than the others, or because a very small minority are substantially wealthier than the others. Because of diminishing returns to additional wives, polygyny will tend to be more widespread in the former scenario.

### 2.2. Social environment

By ‘social environment’ we mean the suite of rules that are superimposed on interaction, including formal laws, (injunctive) social norms, customs and so on. These rules can be deliberately crafted, or can emerge and change autonomously via evolutionary forces of selection (Currie et al., 2021). Adherence can be motivated by specialised enforcers (e.g. by courts) or by other community members. The important common feature is that rules shape the consequences of actions and therefore shape behaviour.

Economists attach immense significance to such rules, seeing their nature as being a primary determinant of a society's material success (e.g. see Acemoglu & Robinson, 2012). Indeed, the very existence of competitive markets relies on rules to, for example, establish and protect property rights. Less appreciated is the central role of rules in governing marriage (Anderson & Bidner, 2023). The requirements of the ceremony, the obligations to kin, the conditions under which divorce is permitted, the associated inheritance rights – all these point to the central role of rules.

The value of having certain rules governing marriage is clear from some of the issues discussed above, including paternity uncertainty and husband commitment. Yet where do rules come from? Scholars have proposed many possibilities (see Acemoglu et al., 2005 for a general taxonomy, and Anderson & Bidner, 2023, for an adaption to the domain of the family) but here we focus on two: efficiency and social conflict.

#### 2.2.1. Efficiency view

The efficiency view sees rules as emerging in order to minimise wasted resources. The efficiency view is natural from an evolutionary perspective: the argument is that selection pressures at the level of the group (e.g. via direct inter-group conflict or differential population growth) will favour groups that use resources more efficiently (Henrich, 2004; Pinker, 2015). Following this line of argument, evolutionary approaches see a rule against polygyny as arising because it ‘is a way of leveling the reproductive opportunities of men, thereby reducing their competitiveness and increasing their likelihood of cooperativeness’ (Alexander, 1987). The argument that monogamy reduces the intensity of male competition is expanded upon by Henrich et al. (2012). They argue that this reduced intensity of competition should manifest itself in lower crime and increases in income per capita (for reasons laid out in Tertilt, 2005). They also argue that monogamy reduces intra-household conflict because of the absence of co-wives, a narrower spousal age gap, greater genetic relatedness within the household and greater paternity certainty. Henrich et al. (2012) and Rexer (2022) present some evidence that is supportive of these predictions that polygyny is associated with more societal conflict. However, other work is not conclusive on this pattern (see, for example, Schacht et al., 2014).

*Insights into the sources of inefficiency:* The evolutionary approaches above already suggest, even implicitly, some descriptions of the competition component that will deliver the group-beneficial properties of monogamy. For instance, competition may unfold by males exerting effort in trying to attract females (much like male choice models, except the female chooses her most preferred male among those competing for her). Male efforts are socially wasteful, so a rule against polygyny may reduce the intensity of competition and thereby allow males to redirect their energies to more productive ends. One could of course append an opportunity for male cooperation (to produce a public good such as defence, say) where willingness to cooperate is jeopardised by competition for wives.

Economists have produced some other possibilities. Even if competition for wives is costless, polygyny produces inefficiencies if males exert effort to engage in extra-marital sex (‘cheating’) and to guard their wives against such attempts of other males (‘guarding’). Simply, polygyny leaves some males unmarried and such males engage in extensive cheating since they have no need to guard. This in turn raises the guarding efforts of those males with wives. The resources devoted to cheating and guarding efforts, at the society level, are a waste and monogamy helps limit such wastage (Francesconi et al., 2016). Parental investment introduces further scope for inefficiency. Marriage itself, viewed as submission to rules that restrict mating opportunities, can improve efficiency because it raises paternity certainty and this increases males’ willingness to invest in offspring (Bethmann & Kvasnicka, 2011; Saint-Paul, 2015).

Economists have been generally sympathetic to the efficiency view and its underlying evolutionary logic. For instance, Alchian (1950) invokes evolution to defend the economists’ assumption that firms maximise profits; firms need only act as if they maximise profits because market competition induces selection on profitability. However, recent work casts doubt on the usefulness of this view given that imposing a rule will generally produce winners and losers. There are various reasons why winners cannot compensate losers, for instance because winners also obtain power and this makes them unable to commit to compensating the losers once the rule is in place and the balance of power has shifted (Acemoglu, 2003). This means that whether a rule is adopted is sensitive to the relative *power* of winners and losers and this leads us to the social conflict view.

#### 2.2.2. Social conflict view

The social conflict view posits that rules are adopted as the result of conflict between different segments of society. A natural social conflict arises from polygyny whereby the poor, weak and wifeless have reason to enthusiastically support rules against polygyny whereas the wealthy, powerful and wived have reason to oppose. The poor and weak are generally in no position to impose society-wide rules, so the emergence of rules against polygyny calls for explanation.

One class of explanation emphasises factors that increase the power of the poor. For instance, industrialisation and the expansion of markets enhance the power of workers, not only because labour is more valuable but also because increased specialisation among workers creates new forms of interdependency across society. Betzig (1986/2017) speculates that these forces ‘may also have brought on reproductive concessions’ by ‘hierarchy heads in positions to make them, in power, legal privilege, productive resources’.

Another class of explanation instead emphasises conflict between powerful groups, such as Church and State. The main argument here is that rules against polygyny are imposed by the Church as part of a larger attempt to limit the accumulation of power, wealth and influence of secular groups (Goody, 1983; MacDonald, 1995; Henrich, 2020; Betzig, 1995).

Once again, these accounts implicitly rely on a competition component (this time between different segments of a society). The plausibility of such accounts depends heavily on the specifics of this competition component, but the intended specifics are easily obfuscated without a formal model. For instance, one may ask why industrialisation brought benefits to the poor in the form of access to wives. Why would higher incomes not be sufficient? Similarly, one may ask why the Church relied on concocting rules of the family to increase their influence, especially given that they were powerful enough to ensure adherence to such rules. We do not doubt that there are satisfying answers to such questions; the point is that a more systematic consideration of the competition component will deliver new accounts of how rules are imposed as the result of social conflict.

*Insights into the dynamics and use of power:* Political economy is a field of economics which is explicitly concerned with, among other things, how groups wield and compete for power. Research in this field is rarely concerned with issues of the family, but the small number of exceptions offers optimism that this could change.

In the context of polygyny, the competition component modelled in Lagerlöf (2010) offers a useful illustration. There are two social groups, elites and non-elites. The elites hold disproportionate resources, but non-elites are able to ‘rebel’ and seize such resources. Polygyny naturally arises as a consequence of resource inequality, leaving some non-elites without a wife. Such a state motivates the non-elites to attack the elites. As a result, the elites impose monogamy rules *upon themselves* as a means to pacify the non-elites. This conclusion bears a surface resemblance to the arguments of Alexander (1987) and Betzig (1986/2017), yet the clarity afforded by a formal model reveals important distinctions. First, the elited are motivated by the immediate and ever-present threat of conflict from within their *own* society; there is no need for external threats, nor for the relatively modern conditions of industrialisation or democracy. Second, there is a clear sense why a rule is required: the non-elites are not pacified by any individual member of the elites eschewing polygyny alone. The elites, being far more populous and decentralised than a despot and his associates, require a coordinated effort. Third, the analysis reveals new insights. For instance, the extent to which the elites are willing to impose a monogamy rule depends on whether the rule is expected to persist in the event that the elites are replaced via rebellion. This result allows for a new perspective on the role of the Church as a means to prolong the longevity of rules (if it is costly for a new ruling elite to switch religions). Finally, the full analysis suggests reasons why the elites pacify the non-elites with monogamy rules rather than, say, resources. For instance, giving resources to non-elites may only enhance their means to mount a successful rebellion.

An alternative approach is to examine the distribution and dynamics of power under democratic conditions. For instance De La Croix and Mariani (2015) develop a model in which rules governing monogamy (and divorce) are sensitive to the relative size of social groups defined by wealth and sex. However, more generally, political economy scholars have thought extensively about how power shifts across groups. For instance Acemoglu and Robinson (2000, 2001) address democratisation, whereby a rich elite gives its political power to citizens. Their key insight is that the elites will sometimes find it optimal to pacify rebellious citizens by granting them political power, as opposed to transfers of resources, because of a commitment problem. Other scholars have sought reasons for why men granted political power (e.g. voting rights) to women. The general insight here is that weak rights for wives harm the interests of males. In particular, this is because weak rights for wives entail insufficient incentives for wives to exert effort (Geddes & Lueck, 2002), insufficient incentives for households to invest in human capital (Doepke & Tertilt, 2009) and an implicit tax on transmitting wealth to daughters (Fernández, 2014). Analyses along these lines hold great promise for generating new hypotheses about rules imposed by social conflict.

One challenge in theorising about rules from the social conflict perspective is that it may not be clear who the relevant powerful groups are. One fruitful way to proceed is to adopt a methodology known as structural estimation. In essence this involves taking a formal model and finding those model parameters which best match real-world data. This then allows the researcher to simulate the effect of any policy. For instance, Tertilt (2005, 2006) develops a rich model of the marriage market, combined with data from sub-Saharan Africa, in order to understand the consequences of banning polygyny in that region. One benefit of this approach is that she is also able to estimate which sub-populations are most likely to gain and lose from a polygyny ban. This provides indirect evidence of which sub-populations are responsible for the absence of rules against polygyny. The analysis reveals that it is older men who have the most to lose from imposing rules against polygyny; among other effects, such a rule lowers the brideprice (which accrues to the older male relatives of the bride). We now turn to a broader discussion of empirical approaches of economists and how these can contribute to the evolution-based perspectives on the family.

## 3. Polygyny: empirical approaches

The empirical work by economists, ultimately concerned with informing contemporary policy, is largely focused on identifying the consequences of polygyny where it is still practised. Although historically pervasive and estimated to have appeared in 85% of societies (Henrich et al., 2012), today polygyny is relatively rare. From a global perspective, it is legally banned in a majority of countries and practiced by only 2% of households (Pew, 2019). That being said, polygyny is still permitted and widespread in many societies across Africa as well as South and West Asia (Anderson & Bidner, 2023; Lawson & Gibson, 2018). According to the most recent Demographic Health Surveys, over a third of women are in polygynous marriages in countries such as Togo, Chad, Senegal, Sierra Leone, Nigeria and Mali.

This focus on estimating the consequences of polygyny for human welfare outcomes is likewise shared by evolutionary social scientists such as Lawson and Gibson (2018), Lawson et al. (2015), Strassmann (1997), Strassmann (2011) and Gibson and Mace (2007), among many others. Because of the emphasis on policy implications, economists endeavour to establish a causal link from polygyny to household behaviour. This primarily implies that they aim to distinguish between causal and selection effects. There are numerous studies establishing an overall negative correlation between the incidence of polygyny and welfare outcomes using large-scale observational data such as poverty, child well-being, household cooperation and intimate partner violence, as well as increased societal conflict. However, an overall positive correlation between child mortality, for example, and the incidence of polygyny could simply emerge because less educated individuals select into polygynous marriages, so that it is not the institution of polygyny per se that leads directly to lower health investments in children, it is instead that less educated parents are less able to care for their children's health. In other words, polygynous household structures do not cause otherwise similarly educated individuals to behave differently. It is rather that lower educated individuals tend to select into polygynous marriages compared with monogamous ones, and this is what is driving the positive correlation between polygyny and child mortality found in the data.

If the positive correlation between child mortality and polygyny is indeed due to these selection effects (by education of parents), then a policy to eradicate polygyny would have no direct impact on child health outcomes. That is to say, although membership in polygynous households may be a way to identify at-risk children, polygyny as a marital institution is unlikely to directly cause child mortality. So, if the focus of policy makers is to improve child health outcomes, resources are better spent on improving women's access to education and public health more generally, rather than prohibiting polygyny.

This focus on causal relationships is directly in line with work outside of economics such as Lawson and Gibson (2018), who emphasise the methodological shortcomings in the literature inferring negative impacts of polygyny on welfare outcomes. The authors highlight the importance of multivariate analysis at the household level where plausibly confounding factors at the community level, such as local socioeconomic and ecological characteristics, are carefully controlled for. The authors give the example from Lawson et al. (2015) who find that the association between polygyny and poor child health found in an aggregated sample of roughly 3000 children, across 56 ethnically diverse Tanzanian villages, disappears after controlling for differences in service provision and rainfall variation at the village level. In their case, the selection effect was that polygyny was more prevalent among certain ethnic groups (such as the Maasai), which are relatively economically disadvantaged for unrelated reasons.

To carefully control for these confounding factors, it is generally necessary to turn to statistical regression techniques that require a sufficiently large number of observations to generate reliable coefficient estimates. The trade-offs from employing this methodology are apparent. On the one hand, relying on larger-scale data sets is often at the cost of potentially relevant context-specific information more available in careful case study analysis. On the other hand, identifying causal channels is of upmost importance, particularly for informing contemporary policy. Subsequent to the ‘identification revolution’, which now characterises most empirical work in economics, economists studying the family tend to focus their efforts on the latter.

In their empirical work, economists endeavour to distinguish between these causal (or direct) effects of polygyny and these alternative selection effects, and they engage with various empirical strategies to this end. In this section, we discuss some of these techniques which are used to understand the consequences of polygyny.

Remaining on the topic of polygyny and child health outcomes, Arthi and Fenske (2018) aim to gain some insight by comparing historical with contemporary data among the Igbo in Nigeria. The benefits to using the historical data include the ability to examine a setting where several of the factors that could confound the links between child mortality and polygyny in the present, such as Christian religious beliefs and attitudes towards modern medicine, are less pronounced. So in this sense, the historical and institutional context are held constant in these regressions. On the other hand, the contemporary data, which include more detailed information at the individual level, allow for a more in-depth investigation of how selection (by individual characteristics) into polygyny plays a role in the association between polygyny and child health.

The authors find an insignificant correlation between child mortality and polygyny in the historical data, but demonstrate a strongly positive and significant correlation (and policy-relevant in magnitude) in the contemporary data. This finding on its own could point to evidence against the institution of polygyny leading directly to poor child health outcomes, as if so, we would expect to also observe the correlation in the historical data. However, we would want to take care in drawing such conclusions given plausible differences in the measurement of child mortality in the historical and contemporary data, and furthermore how reliable historical measures are at all.

The authors move on to explore whether the correlation found in the contemporary data could be explained by selection effects. They first test for which observable characteristics of polygynist parents differ significantly from those of monogamists. They find that children of polygynists are more likely to be born to mothers who are older, less educated, married early, poor and married to older and less educated partners. In a regression analysis, they demonstrate how each of these characteristics is indeed also a significant determinant of child mortality, but when they include them as control variables in the estimations, the significant relationship between polygyny and child mortality remains robust, thus suggesting that these characteristics on their own are not fully explaining the positive correlation between polygyny and child mortality. So although there is evidence of selection effects at play, there is still a statistically robust empirical relationship between polygyny and child mortality in the contemporary context.

Although the contemporary data do include a larger set of control variables, relative to the historical data, there still remain other possible confounding factors that are not included in the dataset, and hence unobservable to the researcher, that could in turn be explaining the relationship between polygyny and child health. To address this concern, the authors compare the estimated coefficient of polygyny (on child health outcomes) in a regression specification with a limited set of control variables to the corresponding estimated coefficient from a specification which includes instead the full set of control variables. If the estimate of the of latter is relatively similar to former, this suggests that the robust positive relationship between polygyny and child mortality is not very sensitive to the inclusion of additional controls. This in turn implies that the coefficient estimate is unlikely to be sensitive to the addition of even more controls, so that adding other unobservable variables into the regression specification is unlikely to alter the relationship. This test demonstrates that selection on additional unobservable variables (which are typically in turn correlated with observable ones) is unlikely to drive the estimated positive relationship between polygyny and child mortality down to zero in the contemporary data.

The above empirical approach does provide evidence to suggest that other unobservable variables, which determine household behaviour and decisions, are unlikely to be confounding the positive relationship between polygyny and poverty outcomes (like child mortality). However, with access to additional data in specific contexts it is possible to explore these relationships much further. For example, using detailed agricultural production data, a few papers have been able to carefully investigate the relationship between polygyny and certain household behaviours in rural settings, while controlling for many economic determinants.

The work of Dessy et al. (2021) investigates risk-sharing arrangements in polygynous families. In particular, they explore how families respond to drought-induced crop failure in rural Mali. The authors seek to identify the causal effect of the interaction of drought incidence and polygyny on crop yields. They posit that polygynous households are more resilient to the adverse impacts of droughts, because they have larger households and hence higher risk-sharing potential. The empirical identification challenge lies with a possible correlation between polygyny and crop output, supposing that polygynous communes tend to locate in more fertile areas, then the authors could be (wrongly) attributing the yield differential as the impact of polygyny, rather than the soil characteristics of the commune. To address this, their linear regression model compares only households residing in the same enumeration area, so that average differences in geographic, economic and cultural factors are held fixed and do not vary over time. In other words, we are comparing yields across monogamous and polygynous households, differentially affected by drought shocks within a small geographical area. The authors find that polygyny has no buffering effects of current droughts on crop yields, but does so with past droughts. They conjecture that because polygyny raises fertility, it enables households in drought-prone rural communities to harness the size and composition of the family workforce to leverage diversification of crop production in the subsequent periods.

To identify causal effects, the above study is able to hold constant the local economic environment in their analysis, and compare outcomes across communities who reside very near to each other. With data on different cropping behaviours within households, an even more detailed analysis can compare activities across plots within families so that even the household-level crop choice and plot characteristics are held constant in the regressions.

An example is Akresh et al. (2012, 2016), who focus in on the implications of polygyny for production efficiency among rural households in Burkina Faso (see also Hidrobo et al., 2021; Damon & McCarthy, 2019). To identify the causal effects, the authors exploit that rural households in this context tend to cultivate several crops on multiple plots, whereby women often have decision-making power over their own plots. Further to this, household members are expected to contribute labour to other household plots. The authors estimate plot yields on a given crop for a given household as a function of plot characteristics and cultivator characteristics. Household cooperation maximises joint farm production and equalises the marginal productivity of inputs across plots controlled by cooperating individuals. The authors therefore consider the yield differences across cultivator pairs to uncover whether polygynous households are better able to cooperate and thus equalise production efficiency across plots. They find that yield differentials between husbands and wives are smaller when the husband has multiple wives. This can be indicative of either greater cooperation among co-wives or between husbands and wives in polygynous households. To differentiate these two mechanisms, the authors can limit their empirical analysis to specific cultivator pairs and they find suggestive evidence that it is improved cooperation among co-wives which is driving the main increase in productive efficiency in polygynous households, compared with monogamous ones. By comparing yields for specific crops, they can rule out selection effects whereby monogamous and polygynous households plant different crops.

Aside from relying on survey data and multivariate regression specifications with carefully defined controls variables, another strategy is to search out sources of variation which are exogenous to the analysis to help to identify the causal mechanisms in question. This is akin to a natural experiment, where we can compare a sample which has been treated with a type of exogenous shock with another sample which remained untreated. With this empirical strategy, there are no unobservable confounding factors associated with this exogenous change in a regression analysis as it is essentially a random event that affects a subset of the population in question.

An example of this type of empirical strategy is used by Heath et al. (2020), who analyse the introduction of a cash transfer programme by the Government of Mali aimed at reducing poverty and improving human capital accumulation. In order to allow for a rigorous impact evaluation, the government collaborated with research partners to implement a two-stage randomised control trial in five regions of the country. A randomly selected subset of communes in the study regions received the programme initially and the remaining set received the programme two years after. So that for two years, there was effectively a ‘treatment group’ (which received the programme) and a control group (which did not). The randomised assignment ensures that baseline characteristics of households across the treatment and control groups are on average the same, so as to minimise selection concerns whereby poorer households (and those that are polygynous) were more likely to be selected into the programme. This implies that the estimated effect of the cash programme on outcomes of interest cannot be due to differences in baseline individual characteristics (i.e. selection effects) and instead is an accurate estimate the programme's direct causal impact.

The authors’ main outcome of interest is women's experience of intimate partner violence (IPV hereafter), and they estimate the impact of the programme on IPV separately for polygynous and monogamous households. Among women in monogamous marriages, they find no statistically significant impacts of treatment on IPV, whereas for women in polygynous marriages, treatment significantly reduces all prevalence and index measures for physical and emotional violence, and controlling behaviours. These findings are more pervasive for second (or later) wives compared with first wives. The authors further uncover that these findings are explained by a larger reduction in men's stress and anxiety, as well as marital disputes, in polygynous households owing to the injection of cash into the households.

The authors also explore whether these findings are explained by observable correlates of polygyny rather than the institution of polygyny per se. To do this, they first assess which correlates appear relevant, by identifying which variables are both correlated with IPV and are significantly different across polygynous and monogamous households. They next assess whether the inclusion of these variables, along with their interactions with the treatment variable, meaningfully changes their estimates of the treatment effect on polygynous households. The results reveal that there is no longer a significant treatment effect among polygynous households when baseline correlates and their interaction with treatment are included. This implies that observable correlates of polygyny, like low education and poverty, largely explain the differential reduction in men's stress and anxiety, with access to increased cash. From a policy perspective, this implies that cash transfer programmes should not necessarily target polygynous households per se, rather they are a good proximate measure for locating disadvantaged households more generally. That what is central is not how the cash transfer interacts with polygynous family structures per se, rather how it interacts with poverty levels which is relevant.

Aside from exploiting exogenous sources of variation imposed by governments or other types of policy actions, it is also possible to rely on exogenous natural sources of unexpected variation like weather shocks. With the increasing availability of geo-located spatial data that can be merged onto household-level surveys, this type of analysis is becoming more common. Rexer (2022) exploits this type of geospatial data to explore the relationship between polygyny and societal conflict to test the hypothesis that societies can be destabilised when a large mass of young men are excluded from the marriage market (Krieger & Renner, 2020; Koos & Neupert-Wentz, 2020; Henrich et al., 2012). The outcome of focus is the number of Boko-Haram deaths within communities across Northern Nigeria. The explanatory variable of interest is a measure of marriage market inequality due to polygyny within a community.

To demonstrate a causal effect from marriage market inequality to conflict, the authors exploit the fact that positive rainfall shocks during girls’ pre-marital adolescence increases marriage inequality in polygynous marriage markets and reduces it in monogamous ones. That is, when young women experience good income realisations in the years before they enter the marriage market, they raise their standards over the types of men they are willing to marry. In polygynous places, women can match with wealthier already-married men, which increases marriage inequality in that locale. The regression estimates demonstrate how an unexpected increase in rainfall during the adolescent period of the average women leads to a differential increase in Boko Haram related deaths annually in polygynous villages. Because the regression analysis exploits the variation in pre-marital weather shocks, and not contemporary shocks (which could impact economic outcomes today and in turn conflict directly), these effects of polygyny on conflict are plausibly causal.

A final empirical strategy used by economists to uncover causal mechanisms is the experimental method, whereby the researcher controls the study environment – so that confounding factors which cause selection effects are eliminated by the research design itself. An example of this type of analysis is Barr et al. (2019), who aim to investigate the inherent dynamics between a husband and co-wives (also see Munro et al., 2019; Rossi, 2019) in polygynous settings. They address this issue directly by implementing carefully designed experimental games among the Nupe in Kwara State, Nigeria. In particular, they invite married adults to play a series of two-person public goods games, with real monetary consequences. They find that polygynous husband–wife pairs contribute significantly less than monogamous husband–wife pairs, and even lower contribution rates among co-wife pairs. They also invite married men and women, from both monogamous and polygynous households, to play the same games against adults from other households. In this case, there are no significant differences across household types. Thus suggesting, that selection effects, whereby less cooperative individuals tend to select into polygynous marriages, are not driving their core findings, and instead there is a causal relationship from polygynous household structures to lower levels of overall cooperation.

The discussion above represents only a small selection of the empirical work by economists on the topic of polygyny. It is meant to highlight the empirical approaches used by economists. What is immediately apparent though is how similar the questions asked in this research are to the focus in other disciplines. In the past, economists have tended to rely on large-scale cross-sectional data to draw conclusions, but over the last few decades with the identification revolution, the work has become more micro-focused and context specific. Further to this, there is a growing appreciation for how relevant the local setting and variation in cultural practices can be for effective policy making (Nunn, 2019). The future cross-discipline synergies appear vast.

## 4. Conclusions

Further integrating the perspectives of economists and evolutionary scholars holds great promise for enhancing our understanding of marriage and the family. Our argument is based on the observation that economics specialises in the analysis of one key Darwinian component – competition – and does so using a distinct methodology that emerges from a concern with contemporary policy issues.

Our argument is not intended to imply that economists are uninterested in evolutionary logic. Indeed, economists have employed evolutionary arguments to foundations of profit maximisation (Alchian, 1950), economic change (Nelson & Winter, 1985), preference formation (Robson, 2001; Becker, 1976; Eaton et al., 2011; Alger & Weibull, 2019), the coevolution of culture and institutions (Tabellini, 2008; Bidner & Francois, 2011; Bisin & Verdier, 2017), economic development (Nunn, 2021) and numerous applications of evolutionary game theory (Young, 1996; Newton, 2018). Similarly, our argument is not intended to suggest that evolutionary scholars are uninterested in contemporary policy; e.g. see Gibson and Lawson (2014, 2015), Lawson et al. (2015) and Lawson and Uggla (2014), Wells et al. (2017) and Tucker and Rende Taylor (2007). Whilst a generalisation, we believe our argument offers a useful starting point for thinking about ways in which closer integration of economics into evolutionary scholarship is likely to be profitable.

We have made our case focusing on the issue of polygyny, but we emphasise that the possibilities are far broader. Our arguments apply to a range of similar issues with disciplinary overlap, which include but are not limited to age gaps between spouses (Bergstrom & Bagnoli, 1993), age at marriage (Wahhaj, 2018), divorce (Hiller & Recoules, 2013), endogamy (Bidner & Eswaran, 2015) and marriage payments (Anderson, 2003; Anderson & Bidner, 2015). For an elaboration upon economic approaches to these and related issues, see Anderson and Bidner (2023).

Although competition is but a single Darwinian component, it is a vital one. The fitness value of a particular trait is not intrinsic; it is entirely dependent upon the nature of competition. A broader appreciation for the various ways that competition can unfold will open more possibilities for understanding the fitness value of traits and thus the evolutionary basis for the outcomes that we observe. This appears particularly promising in the domain of the family and marriage; evolutionary scholars have identified a shift in ‘focus toward the interplay of the marriage system with a broader range of ecological and social factors’ (Fortunato, 2015). We hope to have highlighted specific ways in which economics is well placed to offer insights into such factors.

## Data Availability

N/A.

## Research transparency and reproducibility

N/A.
